# Ergot Alkaloids Produced by Endophytic Fungi of the Genus *Epichloë*

**DOI:** 10.3390/toxins7030773

**Published:** 2015-03-06

**Authors:** Philippe Guerre

**Affiliations:** Département des Sciences Biologiques et Fonctionnelles, Université de Toulouse, INP, ENVT, UR Mycotoxicologie, F-31076 Toulouse, France; E-Mail: p.guerre@envt.fr; Tel.: +33-5-61-19-32-17

**Keywords:** ergot alkaloids, endophytic fungi, *Epichloë*, ergovaline, livestock, toxicology

## Abstract

The development of fungal endophytes of the genus *Epichloë* in grasses results in the production of different groups of alkaloids, whose mechanism and biological spectrum of toxicity can differ considerably. Ergot alkaloids, when present in endophyte-infected tall fescue, are responsible for “fescue toxicosis” in livestock, whereas indole-diterpene alkaloids, when present in endophyte-infected ryegrass, are responsible for “ryegrass staggers”. In contrast, peramine and loline alkaloids are deterrent and/or toxic to insects. Other toxic effects in livestock associated with the consumption of endophyte-infected grass that contain ergot alkaloids include the “sleepy grass” and “drunken horse grass” diseases. Although ergovaline is the main ergopeptine alkaloid produced in endophyte-infected tall fescue and is recognized as responsible for fescue toxicosis, a number of questions still exist concerning the profile of alkaloid production in tall fescue and the worldwide distribution of tall fescue toxicosis. The purpose of this review is to present ergot alkaloids produced in endophyte-infected grass, the factors of variation of their level in plants, and the diseases observed in the mammalian species as relate to the profiles of alkaloid production. In the final section, interactions between ergot alkaloids and drug-metabolizing enzymes are presented as mechanisms that could contribute to toxicity.

## 1. Diseases in Livestock Observed after Consumption of Plants Infected by Endophytic Fungi of the Genus *Epichloë*

Ergot alkaloids are present in various grasses infected by endophytic fungi of the genus *Epichloë* (Table 1) (Note that with a recent taxonomic revision [1] of the genus *Epichloë* has been expanded to include the asexual *Neotyphodium* species known to be derived from sexual *Epichloë* species). The first hypotheses concerning the involvement of ergot alkaloids in livestock health problems that were not linked to the consumption of sclerotia (ergots) of *Claviceps* species was proposed in the 1940s for symptoms observed in cattle fed with tall fescue grass. The description of the disease incudes signs of lameness in winter, usually in the left hind foot, which can lead to loss of the foot [2]. Because the disease occurred in winter, whereas mature *Claviceps* sclerotia are present in the plant in late spring, and because typical signs of toxicity were observed even when the cattle were fed hay that contained no seeds, it was concluded that: “the tall fescue contained some poisonous principle which will cause lameness or shedding of feet similar in all respects to the results from feeding ergot” [2]. After this first description, “fescue foot” was reported in several locations including in Colorado in cattle fed “giant” fescue [3], and in Kentucky in cattle grazing on “Kentucky 31” pastures [4]. Next, the vasoconstrictive properties of extracts of a tall fescue grass obtained from a farm where signs of lameness occurred were demonstrated [5]. The symptoms were then experimentally reproduced in steers fed tall fescue hay [6]. Analysis of the extract of grass revealed the presence of compounds similar to those obtained with extracts of ergots formed by *Claviceps purpurea* on rye, but no ergots were detected by visual examination of the grass [7]. A review of toxicity of tall fescue forage describing the occurrence and severity of fescue foot in several countries pointed to high variability both in the severity and frequency of the syndrome [8].

In the 1970s, several studies were conducted to characterize fescue toxicosis. Changes in blood flow were recognized as being an important mechanism of action of the toxic compounds present in the extract of toxic tall fescue [9]. A “summer slump syndrome” occurring in summer and resulting in decreased performance, was characterized [10]. Also, “fat necrosis” was reported in beef cattle grazing fertilized Kentucky 31 tall fescue [11]. Although analysis of plant extracts provided conflicting results regarding the etiologic agent, the systemic endophyte, *Epichloë coenophiala* = *Neotyphodium coenophialum*, was isolated from tall fescue [12]. Steers fed tall fescue hay infested with *E. coenophiala* showed elevated temperature and decreased performances compared with steers fed hay that was not infested [13]. In 1985, the presence of ergopeptine alkaloids was demonstrated in toxic Kentucky 31 endophyte-infected tall fescue, ergovaline being the most abundant [14]. Ergopeptines extracted from infected tall fescue constricted the dorsal pedal vein of cattle, whereas loline and loline-derivative alkaloids did not [15]. Ergovaline appeared to be the most abundant and the most potent of the ergopeptide alkaloids (see below) and most studies on “fescue toxicosis” reported the ergovaline level in the feed. The toxic threshold of ergovaline was 300−500 µg/kg feed, and cattle appeared to be more sensitive than sheep [16,17].

**Table 1:** Plants infected by endophytic fungi of the genus *Epichloë* known for their toxicity in livestock and horses, and the major alkaloids thought to be responsible.

Grass: Common name	Grass: Latin name	Endophyte	Syndromes or symptoms in grazing livestock	Major alkaloids affecting livestock
Tall fescue	*Lolium arundinaceum (= Schedonorus arundinaceus = Festuca arundinacea)*	*Epichloë coenophiala (= Neotyphodium coenophialum = Acremonium coenophialum)*	Fescue toxicosis, Fescue foot, Summer slump, Fat necrosis ^1^	Ergovaline ^2^
Perennial ryegrass	*L. perenne*	*E. festucae* var. *lolii (= N. lolii = A. lolii)*	Ryegrass staggers	Lolitrem B ^3^, ergovaline
Perennial ryegrass	*L. perenne*	*E. festucae* var. *lolii* x *E. typhina*	Ergot alkaloid toxicity	Ergovaline
Fine fescues	*Festuca* spp.	*E. festucae*	Grazing deterrence	Lolitrem B ^3^, ergovaline
Drunken horse grass	*Achnatherum inebrians (= Stipa inebrians)*	*E. gansuensis* var. *inebrians (= N. gansuense* var. *inebrians*)	Stupor	Ergonovine, ergine
Sleepy grass	*Ach. robustum (= S. robusta)*	*Epichloë* sp.	Stupor	Ergonovine, ergine
Sleepy grass ^4^	*Ach. robustum*	*E. funkii (= N. funkii)*	None reported	Chanoclavine I

^1^: External temperatures play a role in the symptom of the disease: fescue foot is observed in cold winter whereas summer slump syndrome occurs in summer. Fat necrosis is less documented; ^2^: Ergovaline represents more than 80% of ergopeptides in forage grass and more than 50% in seeds. Ergotamine, ergosine, ergocryptine, ergocornine and ergocristine were reported in seeds due to contamination of plant material by *Claviceps* sclerotia (ergots) [18]; ^3^: Lolitrem B and related indole-diterpene alkaloids are recognized as responsible for staggers, whereas the toxic role of ergovaline in endophyte-infected ryegrass is less documented; ^4^ Though generally called sleepy grass, *Ach. robustum* plants with *E. funkii* do not have the toxic effects as those with the other (so far unnamed) *Epichloë* species found near Cloudcroft, New Mexico, USA [19].

Perennial ryegrass has been recognized as responsible for staggers in livestock [20], and endophytic fungi have been described in perennial ryegrass [21]. However, the sclerotia of *Claviceps purpurea* were considered to be causative agents of ryegrass staggers until it was demonstrated that the symptoms cannot be reproduced by feeding sclerotia present in the seed heads [22], and staggers were observed in sheep grazing the base of the ryegrass plant, whereas no signs were observed in sheep that were prevented from grazing the base of the plant [23]. Several tremorgenic mycotoxins of different fungal origin were suspected to be the causative agent of ryegrass staggers until the isolation of lolitrem B [24,25], which appeared to be the main tremorgenic mycotoxin in *Epichloë festucae* var. *lolii* = *Neotyphodium lolii*-infected perennial ryegrass [26]. Ergot alkaloids were also found in these plants, but symptoms of ergot alkaloid toxicity are rarely reported in livestock fed endophyte-infected ryegrass [27]. Because ergovaline often represents 10% to 15% of lolitrem B concentrations [28,29,30,31], it is generally accepted that toxic levels of lolitrem B are reached before toxic levels of ergovaline. For this reason, most studies on endophyte-infected ryegrass has focused on lolitrem B, and little information is available on ergot alkaloids [16,17,32,33], and in particular, nothing is known about possible interactions between ergovaline and lolitrem B.

Because infection of plants by the *Epichloë* species leads to alkaloid production and toxicity in several animal species, a simple solution to avoid toxicity could be to eliminate the endophytes from the grasses sown. However, the mutualistic relationship that exists between the fungal endophytes and their hosts also has several beneficial effects regarding resistance to insects and nematodes, and grass production under stressing conditions [34,35]. *Epichloë*-infected perennial ryegrasses unable to produce lolitrem B were first commercialized under the trade name “Endosafe”. Unfortunately, the use of some of these early cultivars led to ergovaline toxicity in grazing animals. Signs of ergovaline toxicity were unexpected because they had not previously been observed on pastures sown with common endophyte strains, perhaps because symptoms of ergotism had been masked by the signs of staggers [36]. Interestingly, insect feeding studies done with *Epichloë*-infected perennial ryegrass unable to produce ergovaline have demonstrated that this alkaloid was not necessary for deterrence properties [37]. Following these observations, the protocol for evaluation of non-toxic endophyte has changed and the absence of ergot alkaloid production was added to the selection criteria [34,35].

*Achnatherum inebrians* (drunken horse grass; Table 1), associated with rangeland degradation in northern China, has been found to be infected by an *Epichloë gansuensis* var. *inebrians* which produces ergot alkaloids of the simple lysergic acid amide type [38]. Similar alkaloid profiles are observed in some populations of sleepy grass (*Achnatherum robustum*), particularly near Cloudcroft, New Mexico, in the southwestern USA. Livestock and horses that graze these grasses show a profoundly somnolent or stuporous condition [39]. *Agrostis*, *Bromus*, *Elymus*, *Hordeum* and *Poa* species sometimes also contain ergot alkaloids when they are infected by fungal endophytes, but little is known about their toxicity [32,33,40]. In conclusion, a large number of endophyte-infected plants contain ergot alkaloids, but only a few of these associations are known for their toxicity in livestock and horses. The signs of toxicity strongly vary, probably because of the different profiles of alkaloid production.

## 2. Ergots Alkaloids in Plants

Since the discovery of fescue toxicosis, several experimental studies have been conducted to reproduce the disease. In a great number of cases, supplying endophyte-infected tall fescue forage alone failed to induce signs of toxicity, so seeds were added to the feed to increase the ergovaline level [17]. However, adding large amounts of seeds to the feed introduces doubts about the alkaloids responsible for toxicity (see below). At least in some studies, the presence of sclerotia in the seeds was suspected. In addition, the respective percentages of the different alkaloids, especially ergovaline and ergotamine, are not the same in forage plants and seeds, some ergot alkaloids being found only in the seeds [33,41].

The different toxicity of forage used to reproduce fescue toxicosis can also be explained by the stage of maturity of the plant. Indeed, the level of ergovaline in the leaves, stem and inflorescence differs significantly and the stage of maturity of the plant has a major influence on the concentration of ergovaline [42,43,44,45,46]. Three peaks of ergovaline were identified in endophyte-infected tall fescue and endophyte-infected perennial ryegrass in France: the end of spring, the beginning of fall, and mid-winter. In spring, cumulative degree-days of 900 were needed before an increase in ergovaline level occurred both in tall fescue and perennial ryegrass [45,46]. A twofold increase in ergovaline content was observed in a period of only one week at the end of flowering [44,45,46].

Marked variations in the ratio between ergovaline and other alkaloids were also observed in *Epichloë festucae* var. *lolii*-infected perennial ryegrass. A study conducted in endophyte-infected ryegrass straw in Oregon has shown that the concentration of lolitrem B was around tenfold higher than that of ergovaline [29]. By contrast, studies conducted in Europe on wild endophyte-infected ryegrass revealed that the concentration of ergovaline was often higher than the concentration of lolitrem B [47,48]. Genetic factors partly explain these differences, whereby some ecotypes of *Epichloë festucae* var. *lolii* are able to synthesize ergovaline alone, whereas others synthesize lolitrem B alone, but most produce both toxins [47,48,49,50,51]. Also, the environmental conditions during plant growth seem to have a strong influence on the ratio between ergovaline and lolitrem B [46,52,53]. Comparison of New Zealand and German ecotypes have revealed that the environmental conditions during plant growth have a stronger influence on lolitrem B concentrations, than the origin of the ecotype [52]. Similarly, major differences in the concentrations of the ergot alkaloids, ergine and ergonovine, were observed in endophyte-infected drunken horse grass depending on the part of the plant analyzed and stage of maturity [54], whereas temporal variations in alkaloid level was seen in sleepy grass [55].

The levels of ergot alkaloids in plants also vary with several abiotic factors such as temperature, rainfall, fertilization and atmospheric carbon dioxide. Different results were observed depending on the conditions of experimentation and possible interactions between the factors studied. Most of the studies were conducted on endophyte-infected tall fescue and have focused on the effect of nitrogen fertilization. Some studies revealed an increase in ergovaline concentrations when nitrogen fertilization was done, but this effect varied with the dose, the kind of fertilizer and the year of the assay [42,43,44]. The effects of nitrogen fertilization also seem to vary with the part of the plant analyzed, an increase of ergovaline level being observed in the leaves but not in the inflorescence [45]. No correlation was observed between nitrogen input on pastures and ergovaline concentration in straw [56]. Also, the effect of nitrogen input on alkaloid concentrations could vary depending on the cultivar and the alkaloid assayed [57]. An input of phosphorus also increased the level of ergot alkaloids in the plants, but, again, the effect varied with the level of incorporation and the genotype of the endophyte [58]. Analysis of the effect of climate changes on ergot alkaloid contents in endophyte-infected tall fescue revealed that high CO_2_ level decreased ergovaline contents, whereas precipitation had no effect [59]. Also, whereas the levels of alkaloids in the plants showed strong variations depending on the year of analysis, the effect of warming was always the same: it increased ergovaline and total alkaloid levels in fall but had no effect in spring [60].

## 3. Worldwide Distribution

Another question concerns the worldwide distribution of diseases due to ergot alkaloids in endophyte-infected plants. *Epichloë* have been reported in cool season grasses on all continents except Antarctica, but the geographic distribution of cases of toxicity appears to be limited. The distribution of the endophyte-infected plants or the strains of *Epichloë* responsible for the infection explain in part these differences. *Achnatherum robustum*, is limited to southwestern USA. In New Mexico, levels of alkaloids (ergonovine, lysergic acid amide, and isolysergic amide) in sleepy grass populations declined with distance from the Cloudcroft population known for its high toxicity, although *Epichloë*-infection levels increased [55]. This suggests that not all endophyte-infected *Achnatherum robustum* have these alkaloids, as demonstrated in northern New Mexico and southern Colorado where no plants were found to contain ergot alkaloids despite 100% infection [55]. A similar toxicity is observed in animals grazing on endophyte-infected *Achnatherum inebrians* pastures in China. The disease is mainly distributed throughout arid, semi-arid, alpine and subalpine native grasslands of some regions in China, Mongolia and Tibet [54]. High levels of ergonovine and ergine were always measured in plants in areas where the disease was observed [38,54].

By contrast, the worldwide distribution of fescue toxicosis is more difficult to explain. Most cases of toxicity are reported in Australia, USA and New Zealand. In Europe, despite high infection rates of tall fescue by *Epichloë coenophiala* and high levels of ergovaline in plants, only a few cases of toxicity have been reported [61,62,63,64]. Several hypotheses have been proposed to explain these differences. The first concerns the exposure of animals, which is higher in countries where endophyte-infected tall fescue is sown as a monocrop. However, it seems unlikely that no animal is exposed to high endophyte-infected grass in Europe, as demonstrated in the two cases of fescue toxicosis reported in France [61]. The second hypothesis concerns the level of ergot alkaloid in the grass, which may be lower in some regions than in others. These differences could be due to farming practices or climatic factors, which are known to influence the level of ergot alkaloids in whole plants. Studies conducted in endophyte-infected tall fescue in France revealed that an ergovaline level of 300 µg/kg dry matter and more in the whole plant only lasted four weeks, from complete emergence of the inflorescence until the fully ripe stage [45]. The levels of ergovaline in whole plants was lower than levels reported in Georgia, Missouri and Oregon, but the difference was slight [43,44,56]. The third hypothesis concerns the animals. Most studies on tall fescue toxicity report the ergovaline level in feed, but little information is available concerning the amount ingested per kg of body weight, whereas feed intake varies considerably depending on the physiological stage and the animal strain [65]. Genetic diversity has also been proposed to explain some of the differences between strains in their sensitivity to toxicity [65,66].

## 4. Ergot Alkaloids, Not Only Ergovaline

Ergovaline was historically recognized as the main ergopeptide alkaloid, produced by *Epichloë* in tall fescue, and was thought primarily responsible for vasoconstriction and reduced plasma prolactin [67,68]. Most studies on fescue toxicosis have reported the ergovaline level in the feed [17]. However, ergovaline is not the only ergot alkaloid produced, and additive toxicity of other toxins or metabolites is suspected. In a study conducted in sheep using pure ergovaline, the toxin was administered in the feed at a level similar to the one obtained by incorporation of 10% of endophyte-infected tall fescue seeds in the diet [69]. Feed intake, skin temperature, and prolactin were measured as parameters of toxicity. The diet supplemented with the endophyte-infected seeds was more toxic than the diet supplemented with pure ergovaline, suggesting that other compounds present in the seeds have additive toxicity. In another experiment, two diets that contained similar levels of ergovaline, one with seeds of endophyte-infected tall fescue and the other with seeds of endophyte-infected perennial ryegrass, were compared [69]. Again, the toxic effects were more severe in the diet that contained seeds of tall fescue than in the diet that contained ryegrass seeds.

**Figure 1:**
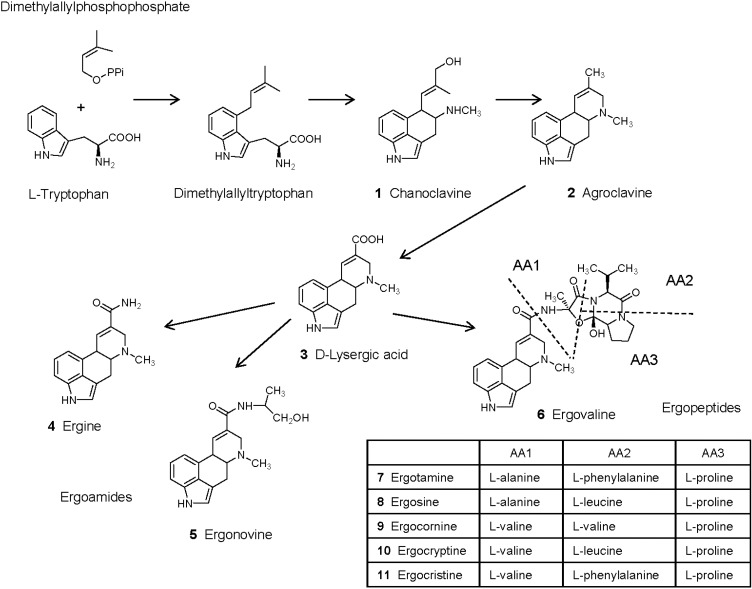
Ergot alkaloids produced by endophytic fungi of the genus *Epichloë* in infected plants (adapted from Shardl *et al.* [70]. Copyright 2006, Elsevier). Ergotamine, ergosine, ergocornine, ergocryptine and ergocristine are additional ergopeptines commonly found in ergots of *Claviceps purpurea* but not *Epichloë* species.

Biosynthetic pathways and genes for ergot alkaloid synthesis in *Epichloë* species have been extensively reviewed in [70,71]. Synthesis of ergot alkaloids begins by the prenylation of l-tryptophan to obtain dimethylallyltryptophan (Figure 1). *N*-methylation and C-oxidation of dimethylallyltryptophan followed by intramolecular rearrangement leads to chanoclavine (1), which can be measured in endophyte-infected *Achnatherum robustum* and has weak binding properties to the dopamine receptors in the brain and no pharmacological effects [19,39,72]. Oxidoreduction of chanoclavine is necessary to obtain agroclavine (2). This compound has antimicrobial and cytostatic properties and is a weak dopaminergic agonist [73,74]. Oxidation of agroclavine leads to the formation of elymoclavine, which is more potent than agroclavine in the prolactin reduction assay [73], and then oxidation of elymoclavine leads to d-lysergic acid (3). d-lysergic acid has been identified in different endophyte-infected plants and in animals fed with endophyte-infected plants [75,76,77]. However, in contrast to observations made on other ergot alkaloids, this compound presented low vasoconstrictor properties in the saphenous vein bioassay and in the right ruminal artery and vein bioassay [78,79,80]. Different amide derivatives of d-lysergic acid (ergopeptines) have been characterized in endophyte-infected plants. Simple amide derivatives are ergoamides whereas more complex derivatives obtained with amino acids are ergopeptines.

Ergine (4, d-lysergic acid amide) and ergonovine (5, d-lysergic acid ethanol amide, also known as ergometrine or ergobasine) are found in endophyte-infected *Ach. inebrians* [38]. However, *E. gansuensis* var. *inebrians* seems unable to produce ergopeptides [51]. Ergine has psychotropic properties whereas ergonovine also has vasoconstrictor properties and reduces the concentration of prolactin, but is less potent than ergotamine [74,81]. In another study, ergonovine did not reduce prolactin and was unable to cause symptoms of fescue toxicosis [82]. Comparison of the vasomotor properties of ergopeptines in the saphenous vein bioassay and in the right ruminal artery and vein ruminal bioassay generally revealed that ergoamides were less potent than ergopeptines, and that their effect was less persistent [79,80,83,84,85]. Also, ergine and ergonovine were less potent than ergovaline in binding to D2-dopamine receptors in cell culture [86]. d-lysergic acid is used for the semi-synthesis of d-lysergic acid diethylamide LSD, which is the most potent hallucinogenic substance known [38,87]. Other effects of LSD include increased arterial blood pressure and reduced prolactin.

The most complex amide derivatives of d-lysergic acid are ergopeptines that are formed with amino acids. Their synthesis involves a two subunit nonribosomal peptide synthetase (lysergyl peptide synthetase subunits LPS1 and LPS2), and sequence variations in LPS1 determine which amino acids are incorporated. Condensation of d-lysergic acid activated by LPS2 with l-alanine, l-valine and l-proline (sequentially added by LPS1) leads to the formation of ergovaline (6). Other ergopeptines produced by *Claviceps* include ergotamine (7), ergosine (8), ergocornine (9), ergocryptine (10), ergocristine (11) [70]. These compounds may be present in some samples of endophyte-infected tall fescue [88], probably due to the contamination of plant material by *Claviceps* sclerotia [18]. Analysis of endophyte-infected samples of tall fescue revealed that ergovaline represented 80%–100% of ergopeptines analyzed in forage grasses and more than 50% in seeds [33]. Ergosine and ergotamine were the other ergot alkaloids found in forage, representing 0%–12% and 0%–6% of the ergopeptines analyzed, respectively [33]. Ergosine, ergotamine and ergocryptine were found in seeds, representing 15%–40% of the ergopeptines analyzed [33,41]. Ergovaline, ergotamine, ergocornine, ergocryptine, and ergocristine triggered contractile responses in the saphenous vein bioassay and in the right ruminal artery and vein bioassay. Ergovaline and ergotamine are generally the most potent, both in terms of intensity and duration of the effect [79,80,83,84,85]. Comparison of the contractile properties of extracts of endophyte-infected tall fescue with ergovaline in these models revealed differential responses to ergot alkaloids in peripheral vasculature and core vasculature [80,85]. Ergovaline, ergotamine, and ergocryptine presented similar binding properties to D2-dopamine receptors in cell culture [86].

Comparisons of the toxic properties of ergot alkaloids should also take in consideration the way they reach their target. Interspecies comparisons of fescue toxicosis revealed that horses are more resistant than cattle and sheep, and that high levels of ergovaline in feed are needed to produce the disease in rodents [17]. Even if several factors explain these differences, pre-hepatic metabolism of alkaloids in the rumen likely influences their toxicity. High metabolism of ergot alkaloids was described in the rumen, and d-lysergic acid was the main metabolite formed [76,77]. However, d-lysergic acid was shown to have less vasomotor activity than ergopetines in several bioassays, and d-lysergic acid was found to be the main metabolite of ergot alkaloids in horses [75]. On the other hand, studies conducted in a parabiotic chamber revealed that the transport mechanism of ergot alkaloids across digestive barriers is an active process, and that this process is more potent via the rumen than via the abomasum [89]. Interestingly, d-lysergic acid and ergonovine had higher absorption potential than ergopeptides, suggesting toxicokinetic differences between ergot alkaloids with possible consequences for toxicity.

Finally, although several ergot alkaloids are present in *Epichloë*-infected plants, it appears that ergoamide derivatives are mainly involved in psychotropic effects, whereas ergopetines are responsible for vasoconstriction and the reduction in prolactin. Comparison of the properties of ergopeptines in different models suggests that ergovaline and ergotamine have the most potent vasomotor properties, and the highest affinity for D2-dopaminergic receptors. Only a few data are available concerning the precursors of d-lysergic acid. Comparison of chanoclavine and agroclavine suggested that the presence of hydroxyl or carbonyl function in the proximate environment of the *N*-methyl of l-tryptophan is necessary to bind to the amine receptor. Oxidation to the carboxylate derivate that leads to d-lysergic acid decreases the binding properties to the biogenic amine receptors.

## 5. Other Effects of Ergot Alkaloids

Because the ergot alkaloids found in *Epichloë*-infected grasses are related to those found in sclerotia (ergots) on florets infected with the *Claviceps* species, most studies of their toxic effects have focused on their pharmacological properties and binding to biogenic amine receptors. However, other mechanisms of action could explain some of the unexpected results observed in fescue toxicosis. Among these mechanisms, interaction of ergot alkaloids with drug-metabolizing enzymes has been suspected. The involvement of drug-metabolizing enzymes in ergot alkaloid metabolism was demonstrated with bromocriptine, a drug synthesized by bromination of ergocryptine. Extensive biotransformation of radiolabeled bromocriptine has been demonstrated in humans, rats, and monkeys, leading to the almost complete absence of the parent drug in urine and bile [90]. Very complex metabolite profiles were observed, with numerous radioactive components; among them, seventeen were identified. Four transformation processes were described, including hydrolytic cleavage of the amide bridge, epimerization, oxidation, and conjugation with glucuronic acid [91]. Even if the specific activity of each metabolite was unknown, these results revealed that the metabolic profile of ergot alkaloids is complex, and that any change in this profile could change toxicity. Interestingly, bromocriptine, in addition to being metabolized by the liver, appeared to be able to induce or inhibit certain drug-metabolizing processes. Studies conducted in the rat revealed that bromocriptine inhibited ethoxyresorufin *O*-deethylase activity, which is considered to be specific to certain cytochrome P450 iso-enzymes [92]. Likewise, bromocriptine enhanced the NADPH cytochrome c reductase activity, which is necessary for the cytochrome P450 monooxygenase system [92]. The first demonstration of the involvement of the cytochrome P450 system in fescue toxicosis was made in sheep. Endophyte-infected fescue hay increased hepatic antipyrine uptake, which was used as an indirect indicator of hepatic mixed-function oxidase activity [93]. This effect was blocked by cimetidine, a cytochrome P450 inhibitor [93]. Interestingly, increased respiration rates and rectal temperatures in animals that had consumed endophyte-infected tall fescue were linked to increased mixed-function oxidase activity, and were partially reversed by cimetidine [93]. A similar effect was proposed to explain the protective effect of an ivermectin treatment on tall fescue toxicosis in cattle [94]. Complementary studies revealed that bromocriptine and other ergopeptide alkaloids are substrates for the CYP3A subfamily of cytochrome P450 [95,96]. d-lysergic acid did not interact with CYP3A, suggesting that it is not a substrate [96]. In humans, inhibition of CYP3A activity by ritonavir resulted in increased ergotamine toxicity [97].

Investigations were conducted to measure the genomic expression of several drug-metabolizing enzymes in rodents. These studies were conducted using high toxic levels of ergovaline by incorporation of endophyte-infected seeds in the feed [98]. Some studies, conducted with animals kept under heat stress, revealed an increase in the expression of CYP2A12, 2D26, 2C13 and 2E1, whereas a decrease was observed in CYP3A25 [99,100]. Other studies conducted at ambient temperature revealed increased expression of CYP1A1, 2C9, 2E1, 3A1 but a decrease in CYP3A7 expression [101]. These effects were observed in parallel with some changes in the expression of the gene-encoding enzymes involved in the mechanisms of defense against oxidative damage [100,101]. Taken together, these results led to the hypothesis that increased expression of cytochrome P450 enzymes and decreased expression of antioxidant enzymes contribute to the toxic effect of fescue toxicosis, especially at high exposure level under heat stress [100,101,102]. Increased oxidation of glutathione in red blood cells was also demonstrated in cattle grazing infected tall fescue under heat stress [103]. The involvement of drug-metabolizing enzyme activities in the susceptibility to fescue toxicosis was also hypothesized in mice selected for their resistance to the disease. Resistant mice displayed significantly higher glutathione-*S*-transferase and uridine diphosphate glucuronosyltransferase activities, and these differences were correlated with toxicity [104]. Interestingly, the glutathione-*S*-transferase activity was correlated with the survival percentage of pups in the resistant line of mice [105]. Sleep time following sodium pentobarbital anesthesia also differed in the resistant and susceptible lines, suggesting differences in cytochrome P450 activity [106]. Differences in the microsomal metabolism of ergotamine were also measured in the two lines challenged with a diet that contained seeds of endophyte-infected tall fescue. The resistant line formed more metabolites than the sensitive line, and a gender difference in the formation of metabolites was also observed [107].

Effects of *Epichloë*-infected hay on the activity of drug-metabolizing enzymes were also measured in sheep. Feeding lactating ewes with an endophyte-infected tall fescue hay that contained 497 µg ergovaline/kg dry matter led to a general decrease in cytochrome P450 enzymes in the liver, with the exception of erythromycin N-demethylase activity, which increased by 330% [108]. Erythromycin is a substrate of the subfamily CYP3A that cross-reacts with anti-human CYP3A4 antibodies [109]. In humans, administration of erythromycin increased the bromocriptine area under the concentration−time curve by 268% [110], whereas administration of ergotamine and ritonavir, a CYP3A inhibitor, led to ergotamine overdosage and toxicity [97]. The cyclic tripeptide moiety of ergopetides was found to be essential for their metabolization by CYP3A. d-lysergic acid did not interact with cytochrome P450 [96]. Comparison of the *in vitro* metabolism of ergotamine performed with microsomes obtained from beef cattle and sheep revealed that both species metabolized the toxin, beef cattle being the most active [111]. Interestingly, breed, gender and species differences have been reported in CYP3A activity in livestock [112]. Taken together, these results suggest that: (i) ergopeptide metabolism by CYP3A may play an important role in tall fescue toxicosis; and (ii) differences in ergopeptine metabolism between species could contribute to the observed differences in toxicity.

In conclusion, ergot alkaloids found in endophyte-infected plants are responsible for various toxic syndromes in horses and livestock. Although several compounds may be present in endophyte-infected plants, few data are available concerning the profile of alkaloid production under field conditions. Ergoamide derivatives are commonly found in endophyte-infected *Achnatherum* spp. plants. The toxicological properties of ergoamides are dominated by psychotropic effects, which are responsible for the symptoms associated with consumption of sleepy grass and drunken horse grass, whereas vasomotor effects are less pronounced. The ergopeptide derivatives are mainly found in endophyte-infected *Lolium* spp. plants, particularly in tall fescue. The toxicological properties of ergopeptines are dominated by their vasomotor effects, which are responsible for “fescue foot” and “summer syndrome”, whose occurrence varies depending on the external temperature. Another effect that occurs before signs of toxicity is a decrease in plasma prolactin concentrations. Ergovaline, which is the most abundant endophyte-derived ergot alkaloid in grass and seeds, was shown in several bioassays to have the highest vasomotor effect. Other effects of ergopeptines include their ability to interact with drug-metabolizing enzymes. In humans, these interactions are well known, and responsible for drug interactions. Genetic differences in drug-metabolizing enzymes have been described in all the animal species. Several studies conducted in rodents and livestock suggest that interactions with drug-metabolizing enzymes occur and may play a role in the toxicity of ergot alkaloids.
